# Adaptive Rewiring in Weighted Networks Shows Specificity, Robustness, and Flexibility

**DOI:** 10.3389/fnsys.2021.580569

**Published:** 2021-03-02

**Authors:** Ilias Rentzeperis, Cees van Leeuwen

**Affiliations:** ^1^Brain and Cognition Research Unit, KU Leuven, Leuven, Belgium; ^2^Department of Cognitive and Developmental Psychology, University of Technology Kaiserslautern, Kaiserslautern, Germany

**Keywords:** structural plasticity, evolving network model, functional connectivity, structure function relation, network diffusion, hebbian plasticity, specificity, robustness

## Abstract

Brain network connections rewire adaptively in response to neural activity. Adaptive rewiring may be understood as a process which, at its every step, is aimed at optimizing the efficiency of signal diffusion. In evolving model networks, this amounts to creating shortcut connections in regions with high diffusion and pruning where diffusion is low. Adaptive rewiring leads over time to topologies akin to brain anatomy: small worlds with rich club and modular or centralized structures. We continue our investigation of adaptive rewiring by focusing on three desiderata: specificity of evolving model network architectures, robustness of dynamically maintained architectures, and flexibility of network evolution to stochastically deviate from specificity and robustness. Our adaptive rewiring model simulations show that specificity and robustness characterize alternative modes of network operation, controlled by a single parameter, the rewiring interval. Small control parameter shifts across a critical transition zone allow switching between the two modes. Adaptive rewiring exhibits greater flexibility for skewed, lognormal connection weight distributions than for normally distributed ones. The results qualify adaptive rewiring as a key principle of self-organized complexity in network architectures, in particular of those that characterize the variety of functional architectures in the brain.

## Introduction

From gestation to termination, the brain continuously undergoes adaptive rewiring; structural changes that shape, maintain, and provide flexibility to function. Adaptive rewiring commonly relies on functional connectivity, i.e., the pairwise statistical dependencies in neural activity patterns (Rubinov et al., 2009; Avena-Koenigsberger et al., 2018). We described adaptive rewiring in a graph-theoretical framework as adding shortcut connections between nodes with strong functional connectivity while pruning connections with weak functional connectivity (Gong and van Leeuwen, 2003, 2004; van den Berg and van Leeuwen, 2004; Rubinov et al., 2009; Jarman et al., 2014; Papadopoulos et al., 2017; Hellrigel et al., 2019).

Whereas those models considered functional connectivity in oscillatory activity, some adaptive rewiring models (Jarman et al., 2017; Rentzeperis and van Leeuwen, 2020) are based on a broader, more abstract notion of neural activity. Heat diffusion on a graph is used to represent the aggregate effects of neural activity, i.e., the traffic of neural signals as a distribution of random walks on the network (Chung, 1996). Experimental studies have shown that heat diffusion models can predict the mass effect of brain activity from anatomical connectivity (Abdelnour et al., 2014, 2018). This motivates our choice of adopting heat diffusion to represent neural mass activity in our current model networks.

Jarman et al. (2017) for binary networks, and Rentzeperis and van Leeuwen (2020) for weighted ones, studied adaptive rewiring based on heat diffusion. By creating shortcut connections in highly trafficked regions and pruning where traffic is low, adaptive rewiring optimizes the network traffic flow. Heat diffusion was used for representing network activity without explicit modeling of input or output; as in our previous models, adaptive rewiring reflects transformations of the system in adaptation to its own spontaneous activity [but see Haqiqatkhah and van Leeuwen (2020) for a model that accomodates input and memory]. The model networks are also undirected and therefore symmetric. This makes them, at best, coarse approximations of the brain at systems level, but reflects the typical use of symmetrical measures such as phase synchrony in large-scale functional connectivity observations.

Notwithstanding these simplifying assumptions, the authors were able to show that adaptive rewiring based on heat diffusion generates complex network connectivity structures akin to those of the brain. Networks evolve small world structures (Sporns and Zwi, 2004; Bassett and Bullmore, 2017) with modular connectivity patterns (Hilgetag et al., 2000; Bullmore and Sporns, 2009) and the rich club effect (Zamora-López et al., 2010; van den Heuvel and Sporns, 2011). In general, a crucial component in the emergence of these connectivity structures is the maintenance of a minimum number of connections among neural units (van den Berg et al., 2012).

Such brain-like structures emerge universally in these models from random initial conditions. Plasticity, however, requires more than just invariably realizing some desirable network features. For instance, when the computational role of a given network changes, the rewiring mechanism should be able to drive the changes that will allow the network to meet the new demands. The demands on plasticity vary, depending on the brain region (Neville and Bavelier, 2000) or the triggering factor, be it development (Sur and Leamey, 2001), learning (Plautz et al., 2000), or recovery following injury (Nudo, 2003, 2013). It is still an open question whether a single adaptive rewiring mechanism is versatile enough to switch upon demand between two different rewiring strategies: either to dynamically maintain an existing functional topology or to modify it.

To address this issue, we introduce the concepts of *specificity, robustness, and flexibility* of evolving network connectivity. *Specificity* means that a network evolves to a connectivity pattern type irrespective of its prior history; for example, an adaptively rewiring network that becomes modular regardless of its current topology -random, modular, or centralized. Biological brain networks show specificity in their evolution and development toward modular structures with dense interconnections within functional units but sparse between different units (Kaas, 2012). Specificity resembles the process of convergence to a global attractor in dynamical systems.

Rewiring shows *robustness* when it maintains a certain connectivity pattern during rewiring, i.e., centralized networks that are adaptively rewired stay centralized and modular ones stay modular. Traditionally, the robustness of a network has been defined as its capacity to withstand node or link failure (Albert et al., 2000; Callaway et al., 2000). Different measures of network functionality, such as connectivity or information spreading efficiency may be used to specify robustness (Bullmore and Sporns, 2009). Bellingeri et al. (2019) have shown that the efficiency of real-world networks depends on the weight distribution of the links. A study on a network's efficiency following rewiring for different weight distributions appears to be a natural continuation of this work. Here, robustness is related to the connectivity pattern of the network after rewiring instead of node or link removal. It is analogous to homeostasis, in that connections are in a dynamic equilibrium that maintains the overall properties of the network. Homeostatic mechanisms are crucial in maintaining the functionality of the brain in the face of constant changes (Turrigiano and Nelson, 2004; Turrigiano, 2012).

A rewiring process shows *flexibility* when it deviates stochastically from the rules of specificity or robustness. Stochastic changes could lead to undesirable noise effects that will degrade the performance of a system. However, when controlled, stochastic deviations could benefit a network's performance in both biological and artificial systems. For instance, randomly rewiring a small subset of connections from a regular network could lead to a significant decrease in the average path length of the network without affecting significantly its connectivity structure (Watts and Strogatz, 1998). Furthermore, a random initialization of the weights of a deep neural network will achieve symmetry breaking, facilitating the convergence of the weights to optimal values during backpropagation (Rumelhart et al., 1985).

We probe the specificity, robustness, and flexibility of our recently proposed adaptive rewiring model (Rentzeperis and van Leeuwen, 2020). During each rewiring step, a pair of connected nodes with low diffusion is pruned while an unconnected pair with high diffusion is connected. The rewiring rule is tuned by a rewiring interval parameter (τ). The value of this parameter is crucial for the resulting type of network. This has been shown when initially random networks are allowed to evolve their structure under repeated application of the adaptive rewiring rule (Jarman et al., 2017; Rentzeperis and van Leeuwen, 2020). For small τ intervals (fast rewiring rates), random networks rewire to become modular; for large τ intervals (slow rewiring rates) they rewire to become centralized (Jarman et al., 2017; Rentzeperis and van Leeuwen, 2020). In a narrow window between fast and slow rewiring rates, we find maximal variability of evolved topologies from highly modular to highly centralized ones.

Evolution of random networks offers only a limited purview on the specificity, robustness, or flexibility of the rewiring process. Rewiring on established connectivity patterns offers a more natural equivalent to brain plasticity. We thus proceeded to probe the rewiring process when the initial networks take on a wide range of pre-established complex connectivity patterns.

Starting from a network with complex connectivity the rewiring process shows different characteristics depending on the value of the rewiring interval. For small rewiring intervals (fast rewiring rates), the rewiring process shows specificity: it reorganizes any pre-established connectivity structure into a modular one. For larger rewiring intervals (slow and intermediate rewiring rates) the process shows robustness: the pre-established type of connectivity structure persists in a dynamic manner. Both robustness and specificity are generally more pronounced for normally than for lognormally weighted networks, with the latter showing a greater degree of flexibility.

## Materials and Methods

The first subsection provides the basic graph nomenclature for defining the networks used. The second introduces heat diffusion, which constitutes the core process behind the rewiring algorithm, and the graph Laplacian, a matrix that captures the structure of the network and controls the diffusion process. The third describes the rewiring algorithm, the fourth the simulation parameters used, the fifth the modularity metric used to characterize the networks' connectivity structure, and the sixth the way the specificity, robustness and flexibility characteristics are found.

### Graph Preliminaries

We define a network as a weighted undirected graph, *G* = *(V, E, W)*, where *V* denotes the set of *N* vertices (nodes) (*V* = {*v*_*i*_|*i* ∈ 1…, *n*}), *E* represents the edges (connections) between them as a set of node pairs (*E* = {(*i,j*)|*i* ∈ *V,j* ∈ *V*}), while the set *W* signifies the strength of the connection, $\left(W=\left\{w^{ij}\in {\mathbb{R}}_{\geq 0}|\left(i,j\right)\in E\right\}\right)$. The cardinalities *|V|* = *n* and *|E|* = *m* refer to the total number of nodes and connections in the network, respectively. A graph *G* can be conveniently described by an *n* × *n* adjacency matrix *A*, with entries showing the strengths of the pairwise connections between nodes, i.e., *A*_*ij*_ = *w*^*ij*^. A zero entry, *w*^*ij*^ = 0, indicates that nodes, *i* and *j*, are not connected. Networks are undirected and with no self-loops, meaning A is symmetric and zero in its diagonal entries (*A*_*ii*_ = 0). For this class of networks, the strength of the nodes is obtained by summing the rows or the columns of *A*, i.e., $s_{j}=\;\overset{n}{\underset{i=1}{\sum }}A_{ij}$. Finally, the degree of a node is defined as the number of edges connected to it, i.e., for a particular node it is the number of non-zero elements in the corresponding row or column of A.

### The Graph Laplacian and the Heat Kernel

The graph Laplacian, *L*, is the graph equivalent of the Laplace Beltrami operator, (∇^2^*f*). Informally speaking, both provide the difference between the average value in the neighborhood of a point and the value of the point. The graph Laplacian features in optimization problems such as graph partitioning (Jianbo and Malik, 2000) and dimensionality reduction (Belkin and Niyogi, 2002). It is defined as *L* = *D–A*, where *A* is an adjacency matrix and *D* is a diagonal matrix containing in its non-zero entries the strengths of *A*: $D_{ii}=\;\overset{n}{\underset{j=1}{\sum }}A_{ij}$.

The normalized graph Laplacian (Chung, 1996) is defined as $L=\;D^{-1/2}LD^{-1/2}$; but if *s*_*i*_ = 0 then $D_{ii}^{-1/2}=0$. Its elements are:

(1) $${ℒ}_{ij}=\{\begin{array}{c} 1\;if\;i=j\\ \frac{-A_{ij}}{\sqrt{s_{i}s_{j}}}\;if\;\left(i,j\right)\in E\;(1)\\ 0\;otherwise \end{array}$$

The normalized graph Laplacian, L, is more suitable than *L* for irregular graphs, i.e., graphs with nodes that differ in degree or strength. This irregularity is captured by the eigenvector $v_{0}={\left[\sqrt{s_{1}},\ldots ,\sqrt{s_{n}}\right]}^{T}$ (Chung and Richardson, 2006) corresponding to its zero eigenvalue, λ_0_ = 0. Since it captures the differences in strength/degree between nodes, the normalized Laplacian is preferred for nearly all real-life graph representations, biological or otherwise. Subsequently, any mention of the graph Laplacian refers to the normalized version L.

The heat equation in a network is defined as:

(2) $$\begin{array}{r} \frac{\partial h\left(t\right)}{\partial t}=\;-Lh\left(t\right) \end{array}$$

The heat kernel, *h(*τ*)*, is an *n* × *n* matrix quantifying the diffusion between all pairs of nodes in the network, i.e., *h(*τ*)*_*ij*_ reflects the amount of heat transferred between nodes *i* and *j* after time τ. The graph Laplacian, L, is of the same size and captures the rate of change of the diffusion.

The unique solution to the heat equation (for unit input from each node) is:

(3) $$\begin{array}{r} h\left(t\right)=e^{-tL}I_{nxn}=e^{-tL} \end{array}$$

We use the exponential part of (3) for diffusion, which indicates the dynamics when we inject unit input to a node (while the rest are zero) for all the nodes in parallel.

### Adaptive Rewiring Algorithm

In the first part of our analysis, the network structure before adaptive rewiring is *G* = G_initial_ with *|V|* = *n* nodes and $\left|E\right|=\left\lfloor 2\frac{\log\left(n\right)}{n}n(n-1)\right\rfloor$ connections, which guarantees networks with random G_initial_ to be connected (Bollobás and Béla, 2001). Adaptive rewiring follows a simple rule: connections with low flow transfer are cut and transferred to non-adjacent nodes with high flow transfer. The rewiring process can be described as follows:

Step 1. Select with uniform probability a node *k* from the nodes with non-zero degree that are also not connected to all other nodes (*k* ∈ *V*| 0 < *d*_*k*_ < *n* − 1).

Step 2. With probability p_random_ select *j*_1_ and *j*_2_ based on the criteria of step 2.1 (random rewiring) otherwise (1- p_random_) select them based on the criteria of step 2.2 (heat diffusion rewiring). Delete the edge (*k, j*_2_) and add the edge (*k, j*_1_). The weight of (*k, j*_1_) is the same as the one of the previously connected edge (*k, j*_2_).

Step 2.1. *j*_1_ is selected randomly from the set of nodes that are not connected to *k*, (*j*_1_ ∈ {*j* ∈ *V*|(*j,k*) ∉ *E*}). *j*_2_ is selected randomly from the set of nodes that are connected to *k*, (*j*_2_ ∈ {*j* ∈ *V*|(*j,k*) ∈ *E*}).

Step 2.2. Calculate the heat kernel, *h(*τ*)*, of the current adjacency matrix *A*, of graph *G*. From the nodes not connected to *k, j*_1_ is the one with the highest heat transfer with *k*. From the nodes connected to *k, j*_2_ is the one with the lowest heat transfer with *k*. Mathematically, this is expressed as follows:

(4) $$\begin{array}{r} j_{1}=argmax_{\left(k,j\right)\notin E,\;k\neq j}h_{kj}\left(\tau \right) \end{array}$$

(5) $$\begin{array}{r} j_{2}=argmin_{\left(k,j\right)\in E,\;k\neq j}\;h_{kj}\left(\tau \right) \end{array}$$

Step 3. Go back to step 1 until *r* edge rewirings have been reached.

In the context of the adaptive rewiring algorithm, we refer to the time variable of the heat kernel as the rewiring interval (τ), since before each rewiring we let the diffusion process for *t* = τ. For small τ values diffusion from each node is contained within a local region; for larger values of τ diffusion is more globally spread. The state of each node, or the amount of heat, can be found by summing the rows or the columns of *h(*τ*)* .

We use networks with two different weight distributions: normal and lognormal. The distribution of neurons' presynaptic weights have been typically modeled as normal, but recent evidence suggests that the weights are skewed, lognormally distributed (Buzsáki and Mizuseki, 2014; Teramae and Fukai, 2014). Normally distributed weights were sampled from the normal probability distribution:

(6) $$\begin{array}{r} p\left(x\right)=\;\frac{1}{\sigma \sqrt{2\pi }}e^{-\frac{{\left(x-\mu \right)}^{2}}{{2\sigma }^{2}}} \end{array}$$

with μ = 1, and σ = 0.25. Negative samples were set to zero. As these are 5 standard deviations away from the mean, their occurrence (one in three and a half million) for all practical purposes did not distort the sampling distribution. Lognormally distributed weights were sampled from a lognormal distribution:

(7) $$\begin{array}{r} p\left(x\right)=\;\frac{1}{\sigma \sqrt{2\pi }}e^{-\frac{{\left(\ln\left(x\right)-\mu \right)}^{2}}{{2\sigma }^{2}}} \end{array}$$

with μ = 0, and σ = 1. In both cases the edges were normalized so that the sum of their weights equal the number of the network's connections. We obtained similar results for different distribution parameters and normalizations. Subsequently, we refer to networks with normally and lognormally distributed weight distributions as normal networks and lognormal networks, respectively.

### Simulation Parameters

**Figure 1:**
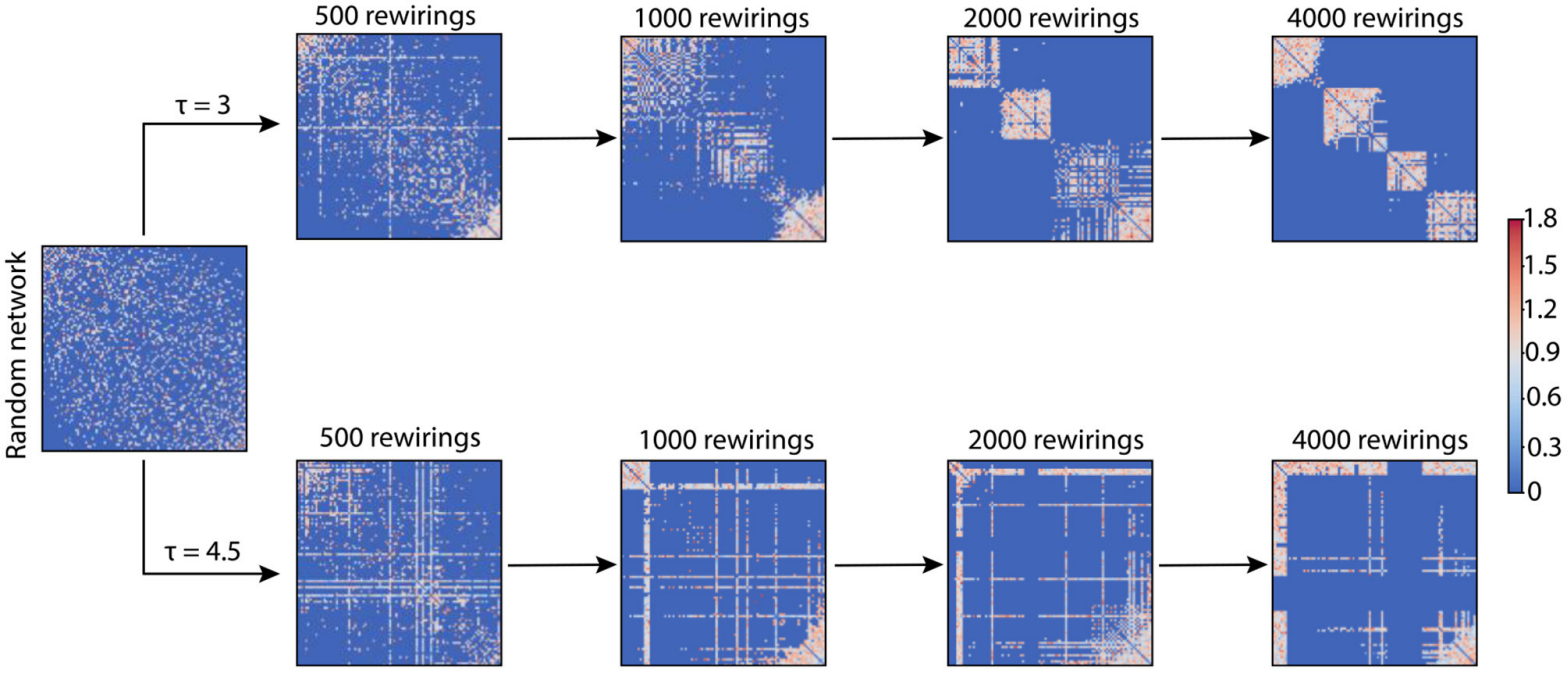
Adjacency matrices for two example networks evolving through adaptive rewiring with different values of τ. The smaller rewiring interval (τ = 3; upper row of adjacency matrices), produces a modular network, i.e., with dense connections within communities and sparse connections between them. The larger rewiring interval (τ = 4.5; lower row of adjacency matrices) has a different effect on the final rewired network; it gives rise to a centralized connectivity structure, where a few nodes have a large number of connections and the rest are sparsely connected or unconnected.

We used networks of 100 nodes with an average degree of 18.24, thus guaranteeing networks with random G_initial_ to be connected. The simulations varied two parameters in the networks: p_random_ and τ. p_random_ was either 0 or 0.2; τ was tested for a wide range of values (τ ∈ [0, 8]). Unless otherwise stated, the number of rewirings we performed in a network was 4,000. Figure 1 shows the evolution of a random network at different stages of rewiring for two different τ values.

### Modularity Measure

In previous studies, we showed that adaptive rewiring leads to networks that are small worlds for all combinations of τ and p_random_, save the degenerative cases (τ close to 0; p_random_ = 1) (Jarman et al., 2017; Rentzeperis and van Leeuwen, 2020). The wide range of rewired networks cannot be distinguished by the small worldness metric. For instance, networks with close small world values could have distant topologies: modular and centralized (Supplementary Figure 1). What distinguishes the aforementioned topologies is the modularity measure.

The modularity measure (*Q*) quantifies the strength of clusters (or communities) within a network; the denser the connections within communities and the sparser between them, the greater *Q* is. *Q* is defined as follows (Newman, 2004):

(8) $$\begin{array}{r} Q=\;\frac{1}{2m}\underset{ij}{\sum }\left[A_{ij}-\frac{s_{i}s_{j}}{2m}\right]\delta \left(c_{i}c_{j}\right) \end{array}$$

where *m* is the sum of all the weights in the network, *s*_*i*_ is the strength of node *i, A*_*ij*_ is the weight of the connection between nodes *i* and *j, c*_*i*_ is the community node *i* is assigned to, and δ*(c*_*i*_,*c*_*j*_*)* is 1 when both nodes *i* and *j* belong to the same community, otherwise it is zero. We used an igraph (Csardi and Nepusz, 2006) implementation of the multilevel algorithm (Blondel et al., 2008), a heuristic modularity optimization function, to assign nodes to communities.

### Specificity, Robustness, and Flexibility

A rewiring process shows specificity if it rewires a network to a specific connectivity pattern irrespective of its initial connectivity. It shows robustness if it does not change the initial connectivity pattern of the network. For a rewired network with an established connectivity pattern, the *Q* value indicates its connectivity state. Large *Q* values are attributed to modular networks and small ones to centralized networks. There is a natural division between modular and centralized connectivity; this division is found at a rewiring interval that imparts maximum variability to the rewired networks, τ_transition_. We use the mean value of the modularity values of the rewired networks at τ_transition_ as this division.

To find the specificity or robustness of the rewiring process we obtain a measure of the relationship between the modularity values of the initial and the rewired networks. We fit a linear function that estimates the modularity of the rewired networks from the modularity of the initial networks. If this line is close to horizontal, the rewiring process shows specificity, if it is close to being diagonal, the rewiring process shows robustness. The greater the variation of the data from the linear fit the more variable the rewiring process is. We call this type of variation flexibility. We quantify flexibility by measuring the squared Pearson correlation coefficient (*R*^2^) of the data. *R*^2^ can be between 0 and 1, the smaller it is the more flexibility the fit shows.

## Results

We first examine the rewiring connectivity patterns emerging for different τ values when the starting network is randomly connected. The final topologies of the networks evolving from initially random networks offer useful demarcations on the rewiring intervals. Specifically, for smaller τ values, the network rewires to be modular and for larger ones centralized. In the next section, we show that for a short window of intermediate τ values the rewired networks show maximum variability in their connectivity patterns: they can be modular, centralized or anything in between. We subsequently probe whether the variability in the initial random networks can explain the distribution of different connectivity structures in the rewired networks. This section is a precursor of the final one where the initial networks have an established connectivity pattern. We show that for larger τ values, the small biases in the connectivity pattern of the starting random networks strongly correlate with the forthcoming patterns of their rewired networks when their weights are lognormally–but not normally–distributed, effectively predicting their variability. We finally probe how the rewiring process affects a network with an already established connectivity pattern. We find that whereas for small τ values the process shows specificity; rewired networks settle into modular connectivity structures -irrespective of their initial connectivity type-, for large τ values the process shows robustness, rewiring leaves the networks' connectivity type intact. In both cases rewiring shows greater flexibility for networks with lognormally distributed weights, compared to normally distributed ones.

### Modular and Centralized Networks

**Figure 2:**
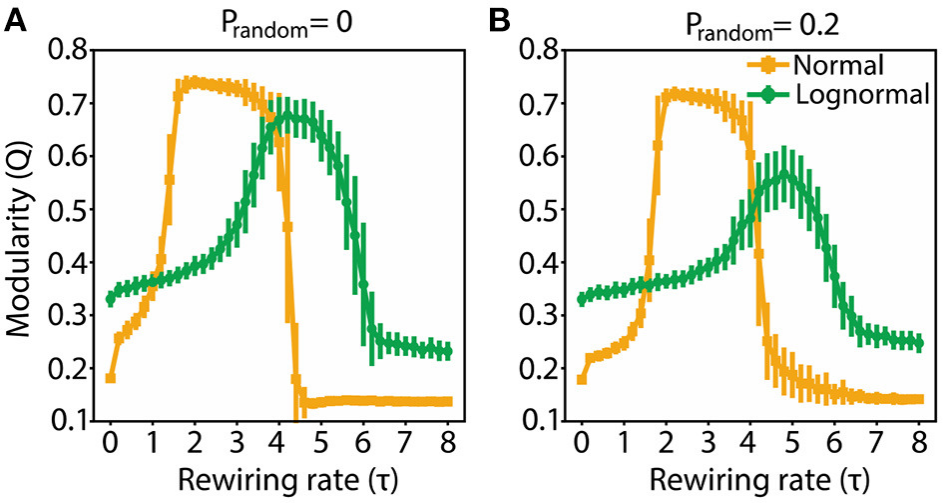
Modularity for both normal and lognormal networks grows as τ increases, but then decreases for larger τ values. **(A)** Q as a function of τ for p_random_ = 0. **(B)** Same as **(A)** for p_random_ = 0.2. Vertical lines indicate standard deviations from 100 instantiations of the rewiring algorithm.

In a modular network, the connections between nodes in a cluster are dense while the connections between nodes in different clusters are sparse. A centralized network is dominated by two sets of nodes: a majority with no or few connections, and a minority with heavy connectivity, the latter acting as a hub. We used the *Q* metric to demarcate the τ values for which the rewired networks are modular (high *Q* values), and the candidate τ values for which the rewired networks are centralized (low *Q* values). The starting network was randomly connected. Both normal and lognormal networks show similar *Q* profiles across τ: *Q* initially increases as a function τ reaching a plateau and then drops off (Figure 2). There are however some differences: normal networks have a wider range of modularity values, reach the plateau faster, sustain it for a broader window of τ values and are more robust to random rewirings compared to lognormal networks (the amplitude drop-off of *Q* with increased randomness is smaller).

A low *Q* value does not guarantee that a network is centralized, since random or close to random networks also have small *Q* values. For a fixed number of total connections, centralized and modular networks have distinct degree distributions: the nodes' degrees for modular networks are close to the mean degree, whereas the ones for centralized networks can deviate significantly. In centralized networks the majority of nodes are sparsely connected, and the rest are densely connected. Hence, compared to modular networks, centralized ones have a larger proportion of nodes with degree outliers. We used the Poisson distribution parameters to derive the number of outliers of rewired networks. The Poisson distribution is a suitable baseline since it is a good fit for the degree distribution of random Erdös–Rényi networks. We considered outlier degrees the ones outside of the range <k> ± 3σ_κ_, where <k> is the mean degree of the network and σ_κ_ the dispersion of the Poisson distribution (σ_κ_ = <k>^1/2^).

**Figure 3:**
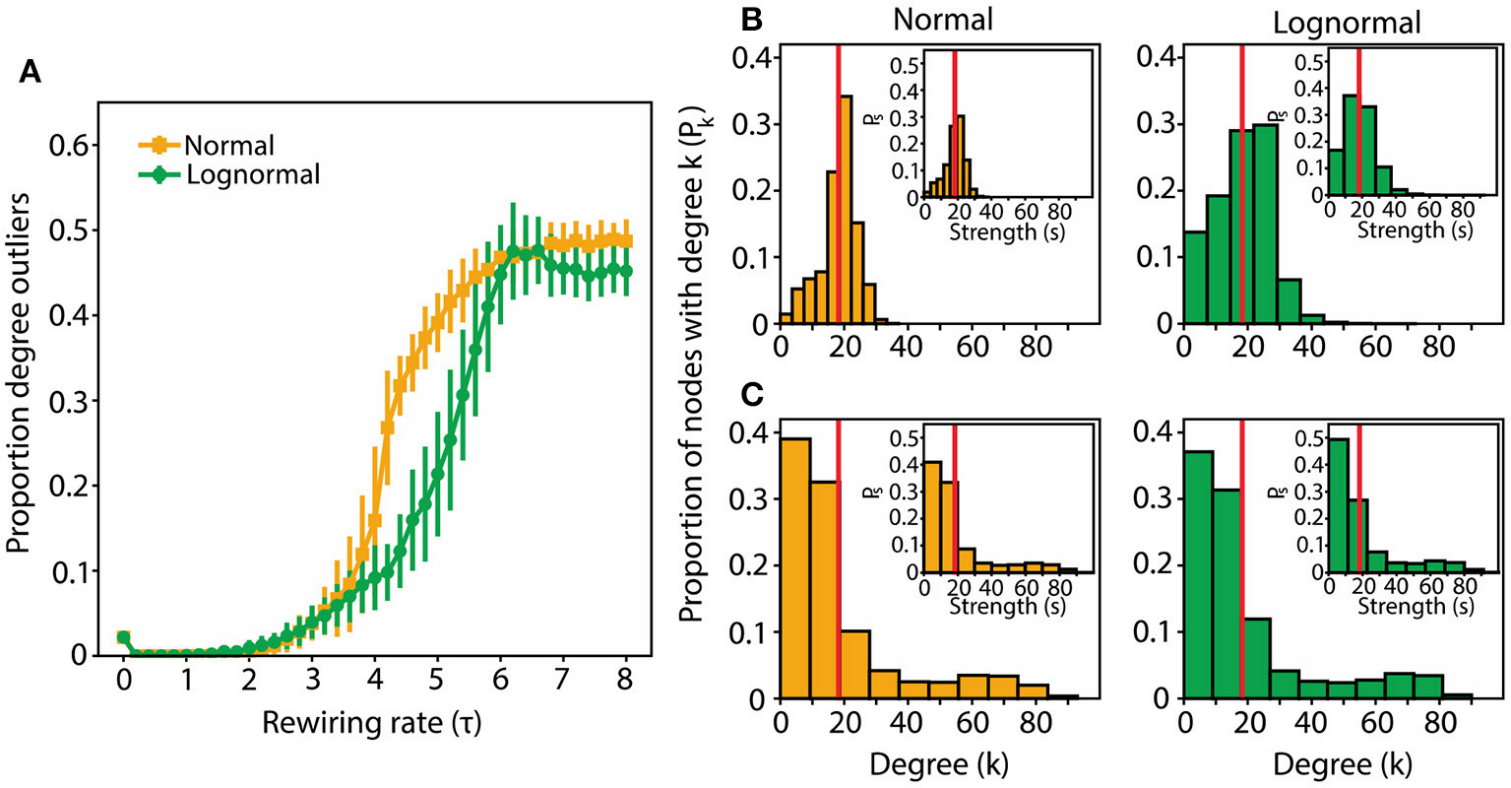
For larger τ values, rewired networks have a large proportion of degree outliers from the mean with the majority of the nodes being sparsely connected and some heavily connected. **(A)** Proportion of nodes with outlier degrees as a function of τ for normal and lognormal networks. Vertical lines indicate the standard deviations from 100 instantiations of the rewiring algorithm. **(B)** The degree distribution for modular normal and lognormal networks (left to right; τ_normal_ = 3, τ_lognormal_ = 4.5). Inset plots show the corresponding strength distributions. **(C)** Same as in **(B)**, but for centralized networks (τ_normal_ = 5, τ_lognormal_ = 7). In all cases p_random_ = 0.2. For **(B,C)** we took the aggregate of 1,000 rewiring instantiations and normalized them so that the sum of the proportions adds to 1.

The proportion of outliers as a function of τ for both normal and lognormal networks follows a sigmoid function (Figure 3A). These results are in agreement with the ones using the modularity measure. Networks with high *Q* values have a small number of outliers and networks with low *Q* values have a large number of outliers (the exception being networks with a τ value close to zero which have small Q values and few outliers; these types are more in line with the properties of a random network). For τ values that give rise to the highest Q values (τ_normal_ = 3, τ_lognormal_ = 4.5) we get networks with degree distributions that are concentrated around the mean (Figure 3B). For larger τ values (τ_normal_ = 5, τ_lognormal_ = 7) that produce networks with the largest number of nodes with degree outliers we get degree distributions with a large spread and a heavy tail (Figure 3C). We get similar strength distributions in the modular and centralized τ ranges (inset plots in Figures 3B,C). Thus, overall with the exception of τ values close to zero and the ones in between a transition range (transition from modular to centralized networks), for small τ values the rewired networks invariably converge to modular structures and for large τ values to centralized structures.

### Network Structure at the Transition Point

**Figure 4:**
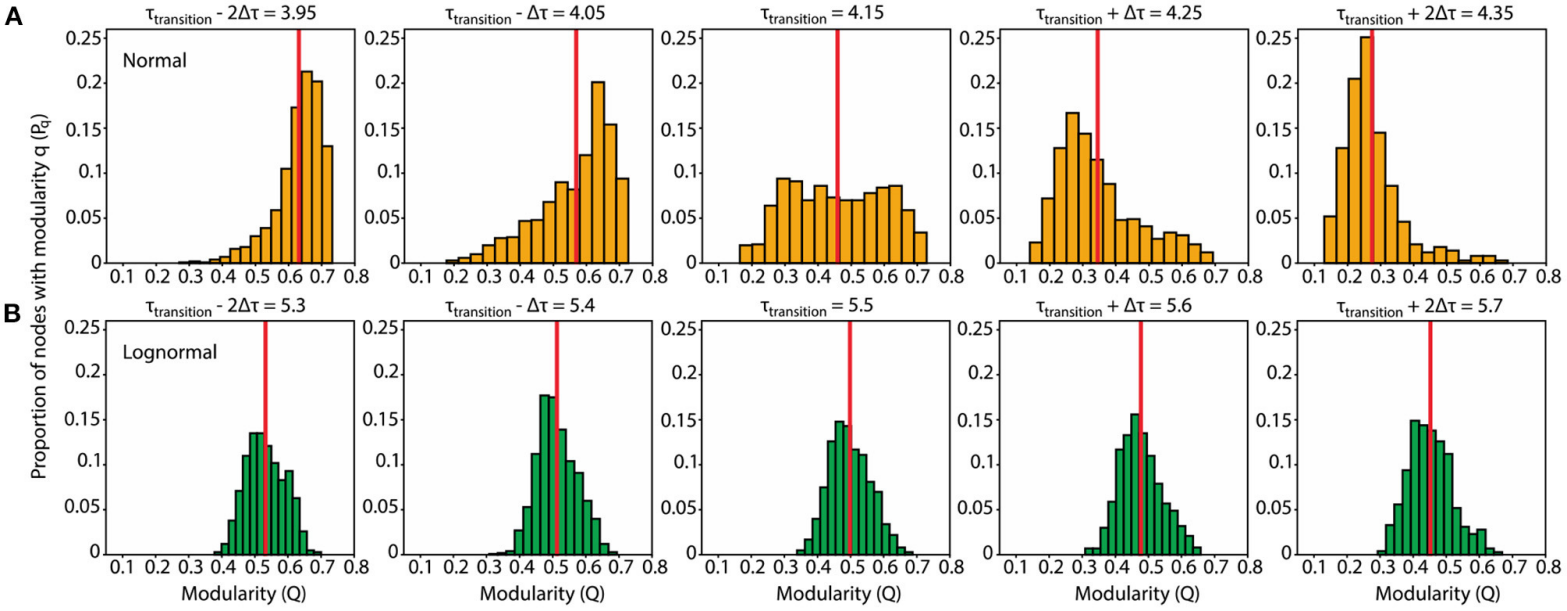
Normal networks show a uniform modularity distribution and are more prone to connectivity transitions after slight perturbations to τ_transition_ compared to lognormal ones which have a modularity distribution with a distinct peak. **(A)** Normal network. Modularity distributions for τ values at and near the transition point: (τ_transition_ – 2δτ, τ_transition_ – δτ, τ_transition_, τ_transition_ + δτ, τ_transition_ + 2δτ) = (3.95, 4.05, 4.15, 4.25, 4.35). For each τ we obtained 1,000 modularity values each corresponding to a different instantiation of the rewiring algorithm **(B)**. Same as **(A)** for the lognormal network τ = (5.3, 5.4, 5.5, 5.6, 5.7).

Our previous analyses showed that, depending on the value of the control parameter τ, the rewiring process would drive networks either to a modular or centralized state. At the boundary between those two states the structure of the network is ambiguous (Figure 4A). Moving the control parameter τ across the boundary causes a phase transition from modular to centralized connectivity. Our aim in this section is to probe the properties of the network at this transition.

We estimated the τ value in the middle of the phase transition from modular to centralized networks (τ_transition_) by locating the inflection point, the point with the largest derivative value, on each of the sigmoid curves representing the proportion degree outliers (Figure 3A). The inflection point for lognormal networks (τ_transition_ = 5.5) is shifted to the right compared to the normal ones (τ_transition_ = 4.15). In a similar analysis on the modularity data, where we located the highest absolute derivative value for the descending part of the modularity curves, we found values close to τ_transition_.

Having established the variability of the rewiring process at τ_transition_, we opted to examine the distribution pattern of the modularity values of the rewired networks. A plausible hypothesis is that the distribution at τ_transition_ is bimodal with one peak centered at the modular region and the other at the distributed one. A contrasting hypothesis is that the distribution is unimodal with the peak in-between the modular and centralized regions. For normal networks, we found a compromise between those two hypotheses: the modularity distribution at τ_transition_ is uniform, with the modularity values on the left and right boundaries giving rise to modular and centralized networks, respectively (Figure 4A, center plot). On the other hand, the modularity distribution for lognormal networks is more in line with the second hypothesis: it is unimodal, and its peak is in-between the modular and centralized regions (Figure 4B, center plot).

The rewired networks at τ_transition_ show maximum variability from highly modular to highly centralized connectivity. Our previous results indicate that beyond certain points to the left and to the right of τ_transition_, variability in the distribution of modularity values will be reduced and modular and more centralized values, respectively, become predominant.

Here we focus on the range around τ_transition_, for which rewired networks show variability. We consider how the distribution pattern of modularity values changes during the transition away from the inflection point and whether these changes are dependent on the weight distribution of the network. We find that, for normal networks, small perturbations (δτ = 0.1) strongly increase the bias of rewiring. Depending on the sign of small perturbations to τ_transition_, the resulting modularity distributions show a distinct peak either at the modular region (negative perturbations; two leftmost plots of Figure 4A) or the centralized one (positive perturbations; two rightmost plots of Figure 4A). In contrast, for lognormal network, changes in response to the same perturbations are much subtler: a mere shift of the peak and corresponding mean of the distribution (Figure 4B).

### Effect of Random Initial Variability on Connectivity Structure After Rewiring

Starting from a random configuration, the rewiring process for a specific τ can result in some variability in the final network, an effect that culminates at τ_transition_ where networks could range from highly modular to highly centralized ones (Figure 4, center plots). Furthermore, at the fringes of its variability range, the initial random network is biased toward a slightly more modular or centralized connectivity pattern. We asked whether these small fortuitous biases in the initial random network are carried over during rewiring, essentially predicting the connectivity pattern of the final network. If this is the case, then some of the variability of the rewired networks could be explained by the variability of the initial network. We tested the hypothesis by comparing the modularity indices of networks before and after rewiring for τ values in the transition point (τ_transition_), and for typical τ values for which the random network rewires to be modular and centralized (τ_modular_ and τ_centralized_, respectively).

**Figure 5:**
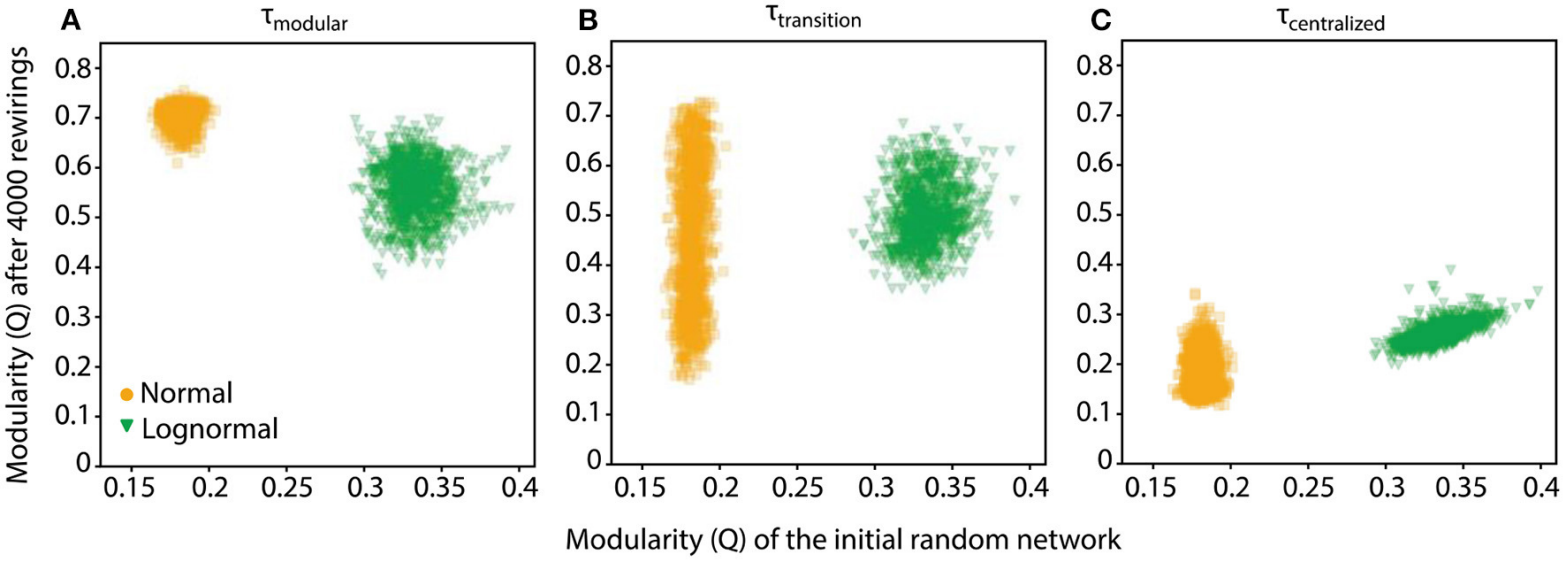
The rewiring process for lognormal networks at τ_centralized_ shows stability. **(A)** Scatter plot of the modularity values, Q, of networks after 4,000 rewirings at τ_modular_ (τ_normal_ = 3, τ_lognormal_ = 4.5) against those of their initial random configuration show no correlation. **(B)** Networks in the transition point (τ_normal_ = 4.15, τ_lognormal_ = 5.5). Scatter plots show weak positive correlation. **(C)** For normal networks in the centralized regime there is still no correlation, however the modularity of the random network for lognormal networks is positively correlated with the final rewired network (τ_normal_ = 5, τ_lognormal_ = 7), with a linear fit of slope 1 and intercept 0.

At τ_transition_, we found very weak correlation between the modulation values of the random and rewired networks ($\rho_{normal}^{transition}=0.12,\;\rho_{lognormal}^{transition}=0.15$; Figure 5B). This indicates that the connectivity pattern of the rewired network at τ_transition_ is nearly independent of the connectivity bias of the initial random network. The same was true for τ_modular_ and τ_centralized_ values for normal networks and for lognormal networks but only at τ_modular_ (Figures 5A,C). At τ_centralized_ lognormal networks showed a strong positive correlation ($\rho_{lognormal}^{centralized}=0.66$, Figure 5C, green data points). The linear fit that best explains the data has a slope of 1 and a bias of 0 which indicates that the rewired network maintained the modularity value it had before rewiring. This suggests that for lognormal networks at τ_centralized_ the rewiring process exhibits robustness. However, random networks exhibit a small range of variability in their topologies and thus any inference about robustness or specificity cannot be conclusive. For initial networks with a wider range of modularity values, rewiring could possibly show different characteristics.

### Specificity, Robustness and Flexibility of Pre-established Complex Network Structures

In the previous section we examined to what extent the biases in the connectivity of a random network would affect rewiring for different τ. Even though an initially random network may serve as a suitable baseline, it has a small range of variability in its connectivity pattern. Hence, it is inconclusive in meriting the specificity, robustness and flexibility of the rewired process. For that, the starting networks to be rewired should have a large variability in their modularity. In this context we ask: Once a network reaches a connectivity pattern, be it modular or centralized, how does it respond to additional rewiring?

To probe this question, we compared the networks' modularity values at 4,000 and 8,000 rewirings. More specifically, an initially random network is rewired 4,000 times at τ_transition_; next, it is rewired an additional 4,000 rewirings at τ_test_. We compare the Q values of the network at 4,000 (at τ_transition_) and 8,000 rewirings (at τ_test_). The set of networks produced at τ_transition_ after the first 4,000 rewirings forms a suitable testbed since the networks have the highest variability and they are also not biased toward a more modular or centralized pattern (Figure 4). We use different τ_test_ values, to probe how they will affect the connectivity pattern of the network established after the first 4,000 rewirings.

We evaluate the relationship between the two sets of Q values by finding a linear fit between them, which is optimal in terms of least squared error. The linear fit takes the form ${\hat{Q}}_{8000}=\alpha Q_{4000}+\beta$, where *Q*_4000_ is a data vector containing the Q values of all the networks after the first 4,000 rewirings, the two scalar terms, α and β, are the slope and bias, respectively, that we adjust to find the fit, ${\hat{Q}}_{8000}$, that gives the best approximation in a least squares error sense of the networks' Q values after an additional 4,000 rewirings. Our classification of networks as modular or centralized is based on the modularity distributions at τ_transition_. A network with a modularity value that is <0.45 is considered modular, one with a modularity value >0.50 is considered centralized. The gray area of modularity values between 0.45 and 0.50 is demarcated by the means from the modularity distributions of lognormal and normal networks at τ_transition_.

**Figure 6:**
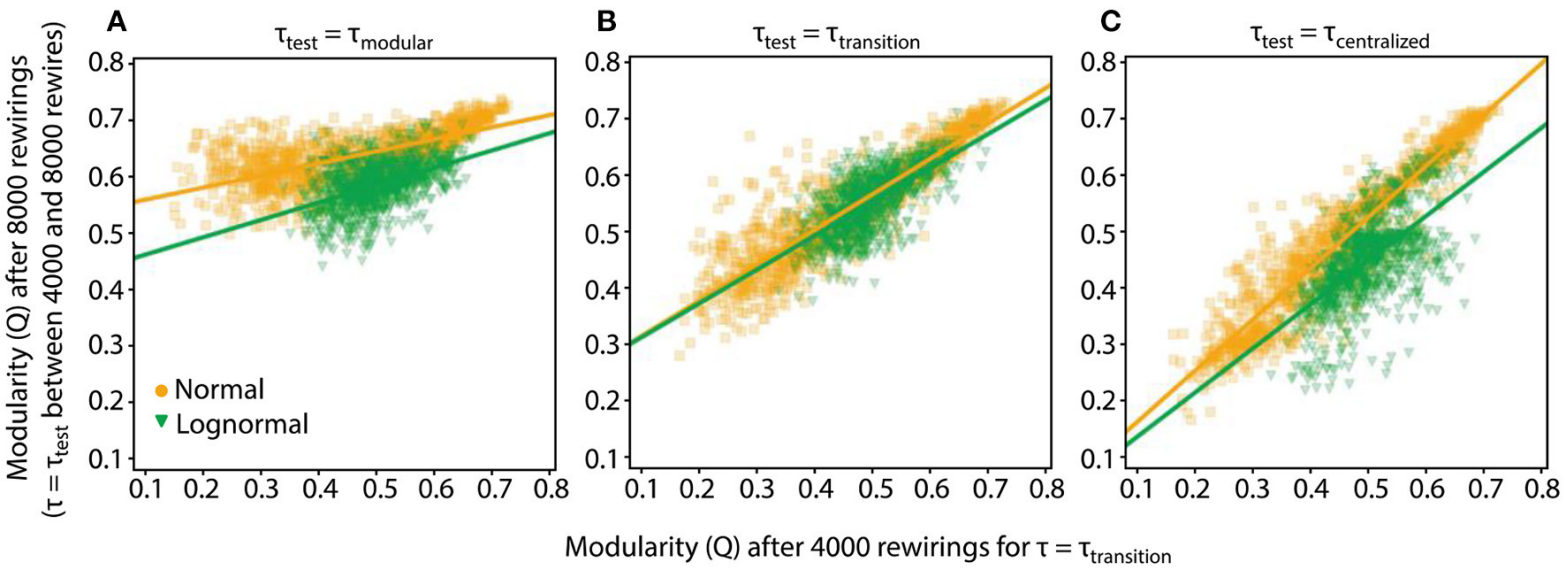
Robustness and specificity of the rewiring process depend on the value of τ_test_. The Q values of networks after 4,000 and 8,000 rewirings along with their linear fits are shown. For the first 4,000 rewirings τ_transition_ (τ_normal_ = 4.15, τ_lognormal_ = 5.5) was used, for the subsequent 4,000 rewirings τ_test_. We collect data from 1,000 rewiring instantiations of a condition. In all cases p_random_ = 0.2. **(A)** τ_test_ = τ_modular_ (τ_normal_ = 3, τ_lognormal_ = 4.5). **(B)** τ_test_ = τ_modular_. **(C)** τ_test_ = τ_centralized_ (τ_normal_ = 5, τ_lognormal_ = 7).

For both normal and lognormal networks at τ_test_ = τ_transition_ (Figure 6B), the estimated slope and bias terms are the same, α = 0.6 and β = 0.25, respectively. This fit indicates robustness, since networks with one kind of connectivity pattern at 4,000 rewirings tend to stay at that pattern at 8,000 rewirings. More specifically, in the centralized range the linear fit gives ${\hat{Q}}_{8000}$ values that are greater than Q_4000_ but still within the centralized range for the most part, i.e., for Q_4000_ values between [0.2, 0.45], the linear fit gives ${\hat{Q}}_{8000}$ values between [0.37, 052]. In the modular range, both Q_4000_ and ${\hat{Q}}_{8000}$ have even closer values, i.e., for Q_4000_ between [0.5,0.7] the linear fit gives ${\hat{Q}}_{8000}$ between [0.55, 0.67].

The previous analysis showed that when τ_test_ is equal to τ_transition_, both normal and lognormal networks sustained on average their connectivity pattern. But for other τ_test_ values, the rewiring process could act differently. One intuitive hypothesis is that for τ_test_ < τ_transition_ and τ_test_ > τ_transition_ rewiring will drive the networks to more modular and centralized connectivity patterns, respectively, i.e., in both cases the rewiring process will show specificity.

This hypothesis holds true when τ_test_ = τ_modular_ (τ_normal_ = 3, τ_lognormal_ = 4.5) since the majority of networks move to modular connectivity patterns, as displayed by their high Q_8000_ values, irrespective of their Q_4000_ values (Figure 6A). This observation is reinforced by the linear fits for both normal and lognormal networks where the estimated bias terms are significantly greater compared to the slopes [(α_normal_ = 0.21, β_normal_ = 0.54) and (α_lognormal_ = 0.31, β_lognormal_ = 0.43)]. For τ_test_ = τ_centralized_ (τ_normal_ = 5, τ_lognormal_ = 7, Figure 6C) the rewired networks do not collapse to a centralized state (small Q_8000_ values) but rather lock in the connectivity state they had at 4,000 rewirings. More specifically, the linear fit for the normal networks and to a lesser extent for the lognormal ones show that the Q_4000_ and ${\hat{Q}}_{8000}$ pairs have similar values for the whole range of modularity values [(α_normal_ = 0.91, β_normal_ = 0.07) and (α_lognormal_ = 0.78, β_lognormal_ = 0.06)].

**Figure 7:**
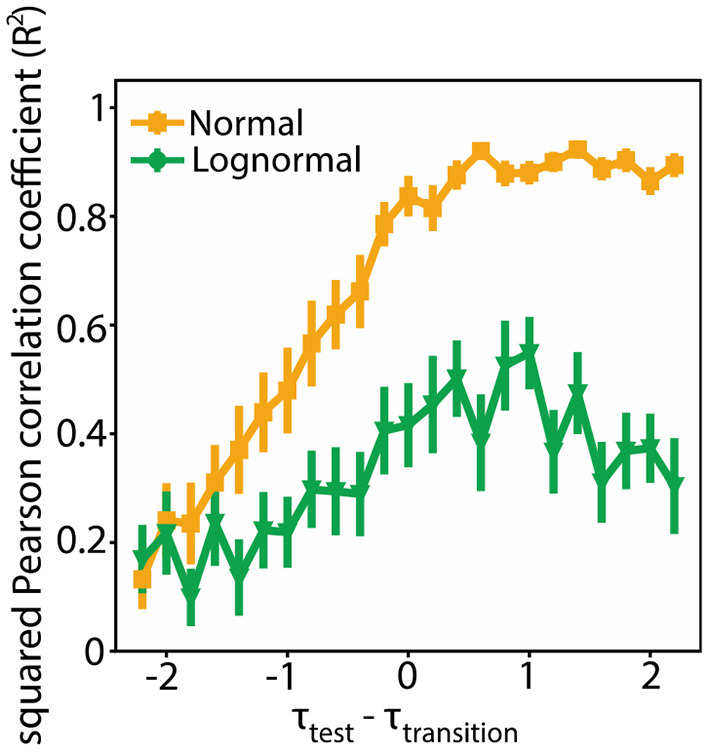
Lognormal networks show greater flexibility compared to normal networks. Squared Pearson correlation coefficient (*R*^2^) between Q_4000_ and Q_8000_ for different τ_test_ values. We used bootstrapping to calculate the mean and standard deviation at each point. More specifically for each point we randomly selected 100 Q pairs (Q_4000_, Q_8000_) with replacement from a sample of 200 pairs and estimated *R*^2^. We repeated this process 1,000 times. We calculated the mean and standard deviation from this 1,000 generated data.

To quantify how consistent the modularity data (Q_4000_, Q_8000_) are with their linear fits we measured their squared Pearson correlation coefficient (*R*^2^). *R*^2^ values close to one merit that a linear fit is a good estimator to the actual data. We found that the *R*^2^ values are smaller for lognormal networks compared to normal ones for the τ_test_ values around τ_transition_ (Figure 7; τ_*test*_ ∈ [τ_*transition*_ − 2, τ_*transition*_ + 2]). In an additional analysis we found that the variance of the fitted slope and bias terms is greater for lognormal networks (Supplementary Figure 2). Both results suggest that there is an inherent flexibility in following a specific linear pattern for lognormal network compared to normal networks. This property imparts them greater flexibility.

## Discussion

Modeling studies for the evolution of complex networks have shown that a simple adaptive rewiring rule provides networks with brain-like, small-world structure. Whereas alternative basic rules, such as winner-take-all or non-linear growth, respectively, lead either to modular (Bauer et al., 2014) or centralized (Bauer and Kaiser, 2017) network structures only, adaptive rewiring can establish both modular and centralized structures, depending on the value of a single control parameter, the rewiring interval (Jarman et al., 2017; Rentzeperis and van Leeuwen, 2020).

Since adaptive rewiring can lead to different architectures, we considered the specificity, robustness, and flexibility of networks evolving through adaptive rewiring. We find specificity of network evolution for adaptive rewiring with small rewiring intervals (fast rewiring rates). That is, networks evolve to be modular irrespective of their previous connectivity state. This result is in line with the underlying architecture of the brain, since many of the brain's functions are broken down into functional units laid out into cortical maps (Mountcastle, 1957; Hubel and Wiesel, 1962). Neurons within functional units are densely connected with each other while connections between units are sparse (Kaas, 2012). This type of organization has been proposed to emerge from an early period of spontaneous activity where wiring is diffuse, followed by mostly sensory dependent activity where the rewiring process is specific within regions (Katz and Shatz, 1996). Here we show, however, that such a distinction in activity is not necessary for modular structure to arise.

The specificity of activity dependent rewiring in the cortex has been amply demonstrated in experimental studies. Notably, in neonatal ferrets, after auditory deafferentation, retinal input was rerouted into the auditory pathway leading to the transformation of neurons in the primary auditory cortex into visually responsive ones, organized in an orientation map comparable to V1 (Sur et al., 1988; Roe et al., 1990; Melchner et al., 2000; Sharma et al., 2000; Horng and Sur, 2006). Thus, activity showed specificity in that it bypassed any structural blueprint of the primary auditory cortex to reorganize it in accord with the novel visual input. Conversely, lack of input activity can also lead to dramatic changes in the reorganization of the cortex as it has been indicated by the effects of sensory loss (Bavelier and Neville, 2002; Merabet and Pascual-Leone, 2010).

Robustness, on the other hand, is involved in maintaining function despite changes taking place in the brain. In the context of our study, it can be viewed as a mechanism that constrains the possible topologies of an adaptable network in that rewiring does not destroy the layout of its overall architecture. We find robustness in networks evolving with larger rewiring intervals (slow and intermediate rewiring rates).

Biological networks are characterized by robustness. They maintain functionality via a range of homeostatic regulatory (Davis and Goodman, 1998) and plasticity (Turrigiano and Nelson, 2004) mechanisms. A number of activity dependent mechanisms are also geared toward homeostasis, such as sliding plasticity thresholds (Bienenstock et al., 1982; Bear, 1995), conservation of total synaptic weight (Royer and Paré, 2003) and spike-timing-dependent plasticity rules (Abbott and Nelson, 2000; for a review Turrigiano and Nelson, 2004; Turrigiano, 2012). Modeling studies have also inferred that homeostatic mechanisms controlling activity within certain boundaries could influence rewiring (Butz et al., 2009; Butz and van Ooyen, 2013). Our results suggest that the brain could rely on adaptive rewiring to retain its functionality because certain of its substructures are in a dynamic equilibrium, that is, connections change adaptively, but macroscopic topological features remain unchanged.

The behavior of the rewiring mechanism in our model is controlled by the rewiring interval. If the rewiring process is poised at the τ_transition_ region, then slight perturbations to τ can sway the rewiring process from robustness to specificity and vice versa. This observation resembles the hypothesis of self-organized criticality, the notion that the brain operates in a boundary between different dynamics (Bak et al., 1987; de Arcangelis et al., 2006; Shin and Kim, 2006; Levina et al., 2007; Wang et al., 2011; Hesse and Gross, 2014). This kind of modus operandi offers a parsimonious explanation on a possible mechanism for the reorganization of the brain that can accommodate specific design principles (specificity) and maintain functionality (robustness). Both specificity and robustness permit stochastic variations deviating from the rule, which generally confer flexibility to biological systems.

The rewiring mechanism shows specificity and robustness for both normal and lognormal networks. However, for lognormal networks, the rewiring process shows greater flexibility in that it steers away from specificity and robustness more often compared to normal networks. Flexibility, as defined here, could enhance adaptability and convergence to optimal values (Rumelhart et al., 1985; Watts and Strogatz, 1998). A possible effect of flexibility in rewiring is a certain amount of diffuse and redundant connections in the brain (Turrigiano and Nelson, 2004). Such connections could facilitate reorganization of the brain during development, in learning, and after injury. In the current adaptive rewiring model, the characteristics of specificity, robustness, and flexibility arise naturally for different rewiring intervals. The versatility of the basic principle embodied by the model, therefore, may underlie the specific mechanisms of brain development, learning, and recovery from injury.

## Data Availability Statement

The original contributions presented in the study are publicly available. The code package replicating the analyses and the Figures is publicly available in a GitHub repository: https://github.com/rentzi/netRewireSpecFlexRob.

## Author Contributions

IR performed and analyzed simulations. CL supervised the project. IR and CL wrote the manuscript.

## Conflict of Interest

The authors declare that the research was conducted in the absence of any commercial or financial relationships that could be construed as a potential conflict of interest.
