# Supplementary figures and images for: Adaptive Rewiring in Weighted Networks Shows Specificity, Robustness, and Flexibility
Source: Front Syst Neurosci. 2021 Mar 2;15:580569. doi: 10.3389/fnsys.2021.580569 (PMC7960922; doi:10.3389/fnsys.2021.580569)

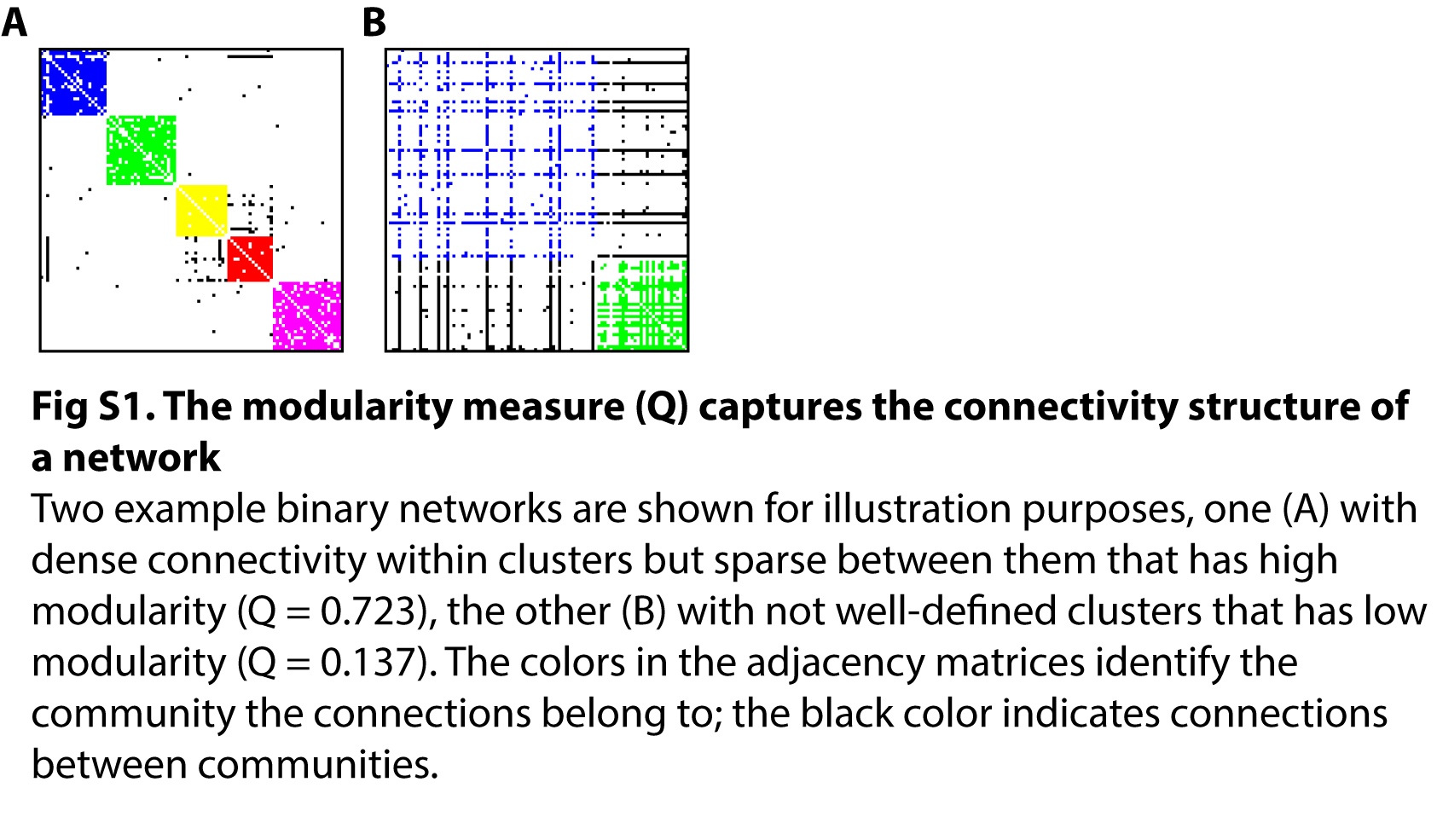

Supplement: Supplementary file 1 [file Image_1.tif]

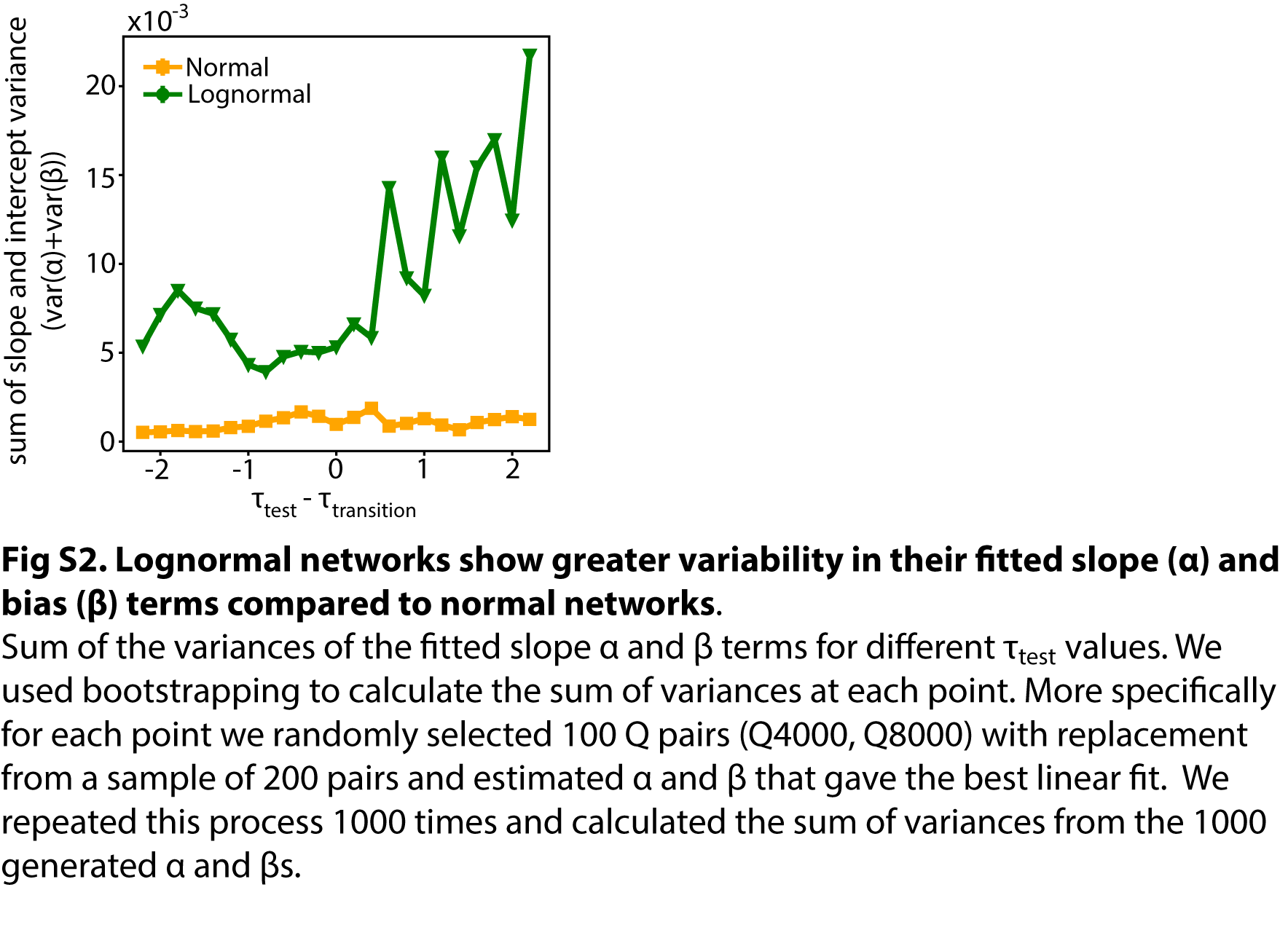

Supplement: Supplementary file 2 [file Image_2.tif]
